# *Tanopicobia* gen. nov., a new genus of quill mites, its phylogenetic placement in the subfamily Picobiinae (Acariformes: Syringophilidae) and picobiine relationships with avian hosts

**DOI:** 10.1371/journal.pone.0225982

**Published:** 2020-01-15

**Authors:** Maciej Skoracki, Bozena Sikora, Leszek Jerzak, Martin Hromada

**Affiliations:** 1 Department of Animal Morphology, Faculty of Biology, Adam Mickiewicz University, Poznań, Poland; 2 Laboratory and Museum of Evolutionary Ecology, Department of Ecology, Faculty of Humanities and Natural Sciences, University of Presov, Prešov, Slovakia; 3 Faculty of Biological Sciences, University of Zielona Góra, Zielona Góra, Poland; Nanjing Agricultural University, CHINA

## Abstract

A new monotypic genus *Tanopicobia* gen. nov. is established for a new species *Tanopicobia trachyphoni* sp. nov., parasitizing *Trachyphonus erythrocephalus* Cabanis, 1878 (Piciformes: Lybiidae) from Tanzania. In phylogenetic analyses based on morphological data and constructed using the maximum parsimony approach, this taxon falls within the subfamily Picobiinae Johnston and Kethley, 1973 in the *Neopicobia*-species-group as closely related to the genus *Pipicobia* Glowska and Schmidt, 2014. *Tanopicobia* differs from *Pipicobia* by the following features in females: genital setae absent; setae *ve* are situated far and posteromedial to the level of setal bases *vi*; setae *3a* are thick and knobbed. Additionally, a new generic key for subfamily Picobiinae is constructed and general host-parasite ecological and phylogenetic relationships are discussed. Picobiines are present in several lineages of neoavian birds, from basal Galloanseres to terminal Telluraves, which are infested by 70 (89.7% of all) species of these ectoparasites.

## Introduction

The subfamily Picobiinae Johnston and Kethley, 1973 (Acariformes: Prostigmata: Cheyletoidea: Syringophilidae) represents taxonomically diverse group of obligate, permanent and highly specialized mite parasites of birds [1], occurring in all zoogeographical regions besides Antarctica [2]. They occupy exclusively short quills (*calamus*) of the contour feathers, except the enigmatic and monotypic genus *Calamincola* Casto, 1977 that is found in the quills of wing feathers of neotropical ani cuckoos [1,3,4]. In this microhabitat, they live, reproduce and feed on soft tissue fluids by piercing the quill wall with their long and styletiform movable cheliceral digits. Only young, fertilized females disperse and infest newly developing quills via a natural opening in the quill wall, “*superior umbilicus****”*** [5]. All representatives of the Picobiinae have a distinctly elongated idiosoma with weakly sclerotized cuticle and relatively short legs, but the unique character differentiating these mites from all other cheyletoid mites (Cheyletoidea) is a physogastry—a phenomenon of enlarged idiosoma in the fertilized females [6,7].

Currently, Picobiinae comprises 81 species grouped in 12 genera (including new genus *Tanopicobia* described herein). They were recorded from about 200 bird species belonging to 11 orders [1,8] (see Table 1), however, only small portion of existing 10,000 bird species was investigated yet, which indicates that the number of species described represents only a small fraction of the actual picobiine diversity.

**Table 1:** The genera of the subfamily Picobiinae with their host distribution (on the order level).

Picobiine mite genus	No of species	Host order
*Calamincola* Casto, 1978	1	Cuculiformes
*Charadriineopicobia* Skoracki, Spicer and OConnor, 2014	3	Charadriiformes
*Columbiphilus* Kivganov and Sharafat, 1995	4	Galliformes, Pterocliformes
*Gunabopicobia* Skoracki and Hromada, 2013	2	Columbiformes
*Lawrencipicobia* Skoracki and Hromada, 2013	1	Psittaciformes
*Neopicobia* Skoracki, 2011	10	Passeriformes, Piciformes
*Phipicobia* Glowska and Schmidt, 2014	1	Passeriformes
*Picobia* Heller, 1878	40	Bucerotiformes, Passeriformes, Piciformes
*Pipicobia* Glowska and Schmidt, 2014	4	Passeriformes, Psittaciformes
*Pseudopicobia* Skoracki, Scibek and Sikora, 2012	3	Galbuliformes
*Rafapicobia* Skoracki, 2011	11	Gruiformes, Passeriformes, Piciformes, Psittaciformes
*Tanopicobia* **gen. nov.**	1	Piciformes

Picobiines are represented mostly by monoxenous species (56%) [1]. Oligoxenous or mesostenoxenous species (restricted to one genus of the host or one family of the host) constitute 18% and 22%, respectively, of whole picobiine fauna. The smallest number (only 4%) is represented by metastenoxenous species associated with hosts from more than one family but restricted to one order of the avian host [1]. At the generic level, the host specificity of picobiines is still significant. Seven picobiine genera (58%) parasitize birds of one order, remaining five genera (42%) are associated with birds of two to four orders (Table 1).

Present paper has several aims: i) a new genus is proposed for a new species *Tanopicobia trachyphoni* gen. et sp. nov., associated with the red-and-yellow barbet *Trachyphonus erythrocephalus* Cabanis, 1878 (Piciformes: Lybiidae) in Tanzania, ii) to construct a new generic key for subfamily Picobiinae, iii) to reconstruct the phylogeny of the subfamily and iv) to discuss host-parasite relationships and possible co-phylogeny.

## Material and methods

The mite material used in this study was collected from dry bird skins deposited in the Bavarian State Collection of Zoology, Munich, Germany. Quills of contour feathers were examined using a dissecting microscope and opened with a fine scalpel. Before mounting, all collected mites were softened and cleared in Nesbitt’s solution at 60°C for 12 h [5]. Identifications and drawings of mite specimens were carried out with a ZEISS Axioscope (Carl-Zeiss AG, Germany) light microscope equipped with DIC optics and camera lucida. In the descriptions below, the idiosomal setation follows Grandjean [9] as adapted for Prostigmata by Kethley [10]. Nomenclature of leg setae follows that proposed by Grandjean [11]. Morphological terminology follows Skoracki [5]. All measurements are in micrometers (μm). Measurement ranges for paratypes are given in brackets following the data for a holotype. Common and scientific names of the birds follow Clements et al. [12].

### Phylogenetic analysis

Although the main goal of the study was to examine relationships at the generic level, all operational taxonomic units (OTUs) were represented by taxonomic species (morphospecies) in the cladistic analysis. Picobiine genera were represented by one species of each genus (S1 Table). *Syringophilus bipectinatus* Haller, 1880, representing the second syringophilid subfamily–Syringophilinae Lavoipierre, 1953, was used as an outgroup. Because each particular picobiine genus is represented by a single species in the present analysis (S1 Table), character states appearing as autapomorphies represent true synapomorphies for genera. The autapomorphic characters included in the analysis are essential for generic diagnoses and useful for future phylogenetic studies at lower taxonomic levels [13].

A total of 13 OTUs and 32 morphological characters (among them 15 autapomorphies) were included in our data matrix (S1 and S2 Tables). A detailed discussion of the morphological characters used in the present study is provided by Skoracki [5] and Skoracki et al. [1]. Constructing of the taxa matrix was done using NEXUS Data Editor 0.5.0 [14]. Analyses of character distribution on trees were performed in WINCLADA [15]. Only qualitative, unordered, and *a priori* unweighted characters were used in analyses. We applied a multistate contingent coding strategy, [16] which is considered as the most useful among available approaches [17]. Following this strategy, characters having multiple states were interpreted as unordered and were not modified into binary characters. In the data matrix, missing states were coded as "?" and inapplicable as "-".

Reconstruction of phylogenetic relationships was performed with PAUP 4.0 beta version for IBM [18] in conjunction with PRAP2 [19] to conduct a ratchet analysis (1000 iterations; 10 random cycles, collapsed zero-branches in effect; options are the default). Nodal support was evaluated by Bremer indices calculated with PRAP2. Analysis of character distributions, drawing, and editing of the trees were performed in TreeView 1.5.2. [20].

### Visualization of host phylogeny

To visualize host phylogeny, a tree was constructed based on consensus avian phylogenetic tool available at http://birdtree.org/ [21]. For each bird order, host species of the first picobiinae mite found out in particular host order (see [2]) was used in the analysis. As the source of data, we used the “Hackett All Species tree” with 1000 randomly generated trees. Currently, this tool is widely used in bird evolutionary ecology studies (e.g., [22,23], including the investigation the host phylogenies of bird parasites [7,24]). The most credible tree was then determined using the tool TreeAnnotator v1.8.2 in the software BEAST v1.8.2 [25]. The consensus tree was then graphically adjusted in FigTree v1.4.2 [26].

### Bipartite networks and statistics

To visualize pattern in studied parasite-host ecological web, we used a “bipartite” package for R [27]. A number of picobiine mite species infesting each bird order were used as a quantitative indices (Table 1).

### Nomenclatural acts

The electronic edition of this article conforms to the requirements of the amended International Code of Zoological Nomenclature, and hence the new names contained herein are available under that Code from the electronic edition of this article. This published work and the nomenclatural acts it contains have been registered in ZooBank, the online registration system for the ICZN. The ZooBank LSIDs (Life Science Identifiers) can be resolved and the associated information viewed through any standard web browser by appending the LSID to the prefix "http://zoobank.org/". The LSID for this publication is:

urn:lsid:zoobank.org:pub:CB6C15E1-5608-4404-8FEB-3001C8D366A4. The electronic edition of this work was published in a journal with an ISSN, and has been archived and is available from the following digital repositories: PubMed Central, LOCKSS.

## Results

### Systematics

#### *Tanopicobia* gen. nov.

urn:lsid:zoobank.org:act:F8D9B35B-3977-472A-9B5C-A3C20E95BA55

**Fig 1:**
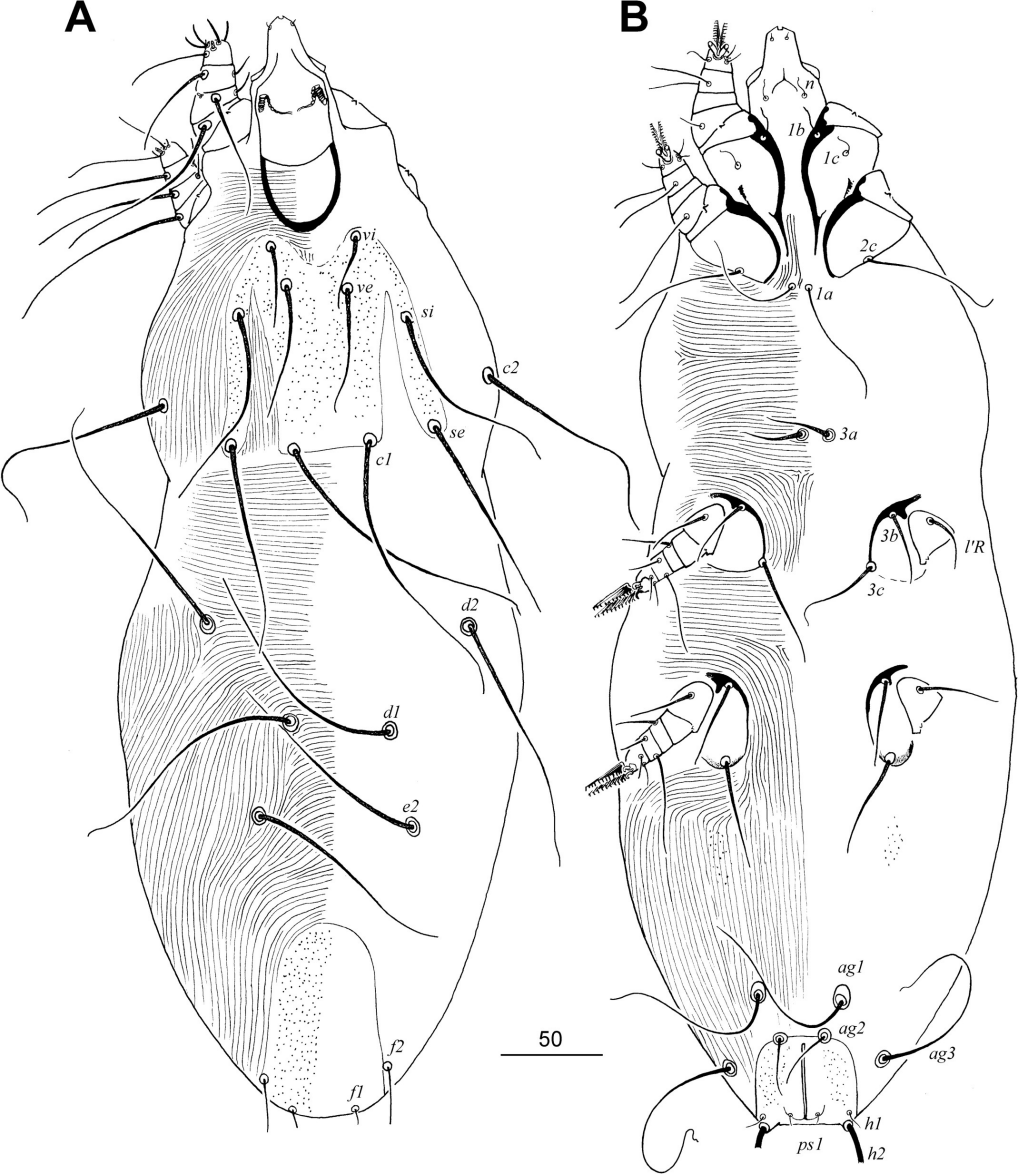
*Tanopicobia trachyphoni* gen. et sp. nov. Female. A, dorsal view; B, ventral view.

**Fig 2:**
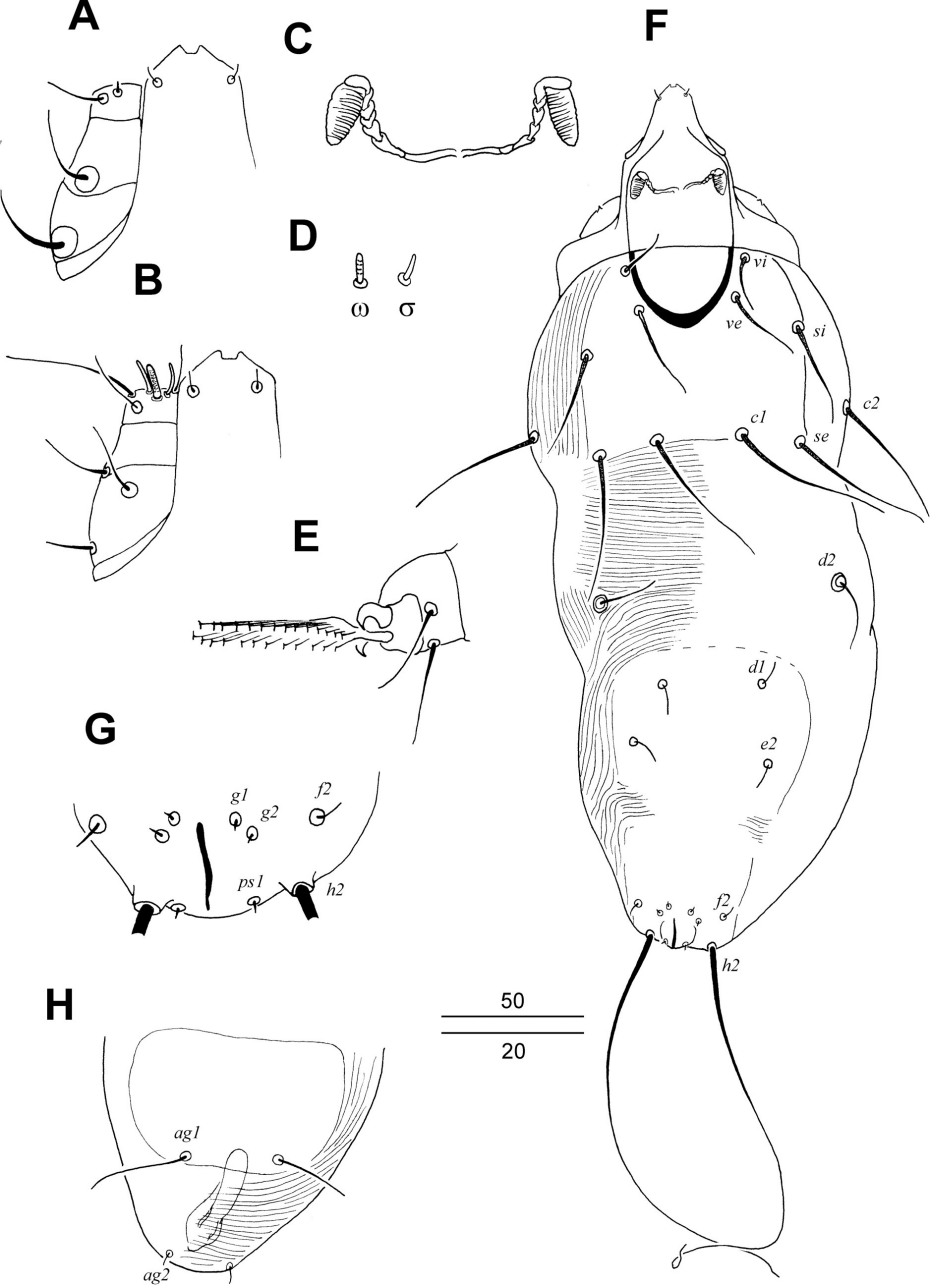
*Tanopicobia trachyphoni* gen. et sp. nov. Female. A, hypostomal apex and palp in dorsal view; B, apex and palp in ventral view; C, Peritremes; D, genito-anal region; E, solenidia of leg I; F, tarsus of leg III. Male. G, dorsal view; H, genito-anal region; I, opisthosoma in ventral view.

**Diagnosis.** FEMALE (Figs 1 and 2). Hypostomal apex tapering. Peritremes mouth-shaped, lateral branches with short and ill-visible borders between chambers. Setae *ve* situated far and posteromedial to level of setae *vi*. Propodonotal shield entire, M-shaped. Opisthonotal and genital lobes absent. Bases of setae *1a* separated. Genital setae absent. Pseudanal series represented by 1 pair of setae. Apodemes I with small thorn-like protuberances. Solenidia phi (*φ*) on tibiae I absent. Physogastric form with weakly enlarged idiosoma.

MALE (Fig 2). Features as in females except: propodonotal shield rectangular in shape, and genital series represented by 2 pairs of setae.

**Type species.** *Tanopicobia trachyphoni* sp. nov.

**Host range.** Piciformes (Lybiidae).

**Distribution.** Afrotropical region (Tanzania).

**Differential diagnosis.** This new genus is closely related to *Pipicobia* Glowska and Schmidt, 2014 which representatives are associated with passerines (of the families Acanthizidae, Locustellidae, Monarchidae) and parrots (Psittaculidae) [1,28]. In females of both genera, the hypostomal apex is tapering; the peritremes are mouth-shaped and with ill-visible borders between chambers in lateral branches; the opisthonotal and genital lobes are absent; bases of setae *1a* are separated; the apodemes I are with small thorn-like protuberances; solenidia phi (*φ*) on tibiae I are absent; and physogastric form is with weakly enlarged idiosoma.

*Tanopicobia* gen. nov. differs from *Pipicobia* by the following features: in females of *Tanopicobia*, bases of setae *ve* are situated far and posteromedial to the level of setal bases *vi*; setae *3a* are thick and ornamented; genital setae are absent. In females of *Pipicobia*, bases of setae *vi* and *ve* are situated in close proximity, and *ve* are situated postero- or posterolateral to the level of setal bases *vi*; setae *3a* are thin and similar to *1a*; genital setae are present.

***Etymology*.** The name of this genus is a compilation of the “*Tano*”–African river god of war, one of the sons of main Akan deity Nyame, and “*Picobia*”–type genus for the subfamily Picobiinae.

#### *Tanopicobia trachyphoni* sp. nov.

urn:lsid:zoobank.org:act:B977A54E-B8E9-40DC-9A90-AC1C17FBFB9A

**Description.** FEMALE, holotype and two female paratypes. Total body length of holotype 535 (550). *Gnathosoma*. Infracapitulum and stylophore apunctate. Each medial branch of peritremes with six chambers, each lateral branch with weakly marked borders between chambers. Movable cheliceral digit edentate on proximal end. *Idiosoma*. Setae *vi*, *ve*, *si*, *se*, *c1*, *c2*, *d1*, *d2*, *e2*, *3b*, *4b*, *3c*, *4c*, and *3a* strongly ornamented. Setae *1a* and *ag1–3* smooth. Propodonotal shield densely punctate, bearing setae *vi*, *ve*, *si*, *c1*, and *se*. Length ratio of setae *vi*:*ve*:*si* 1:1.8–2:2.4. Hysteronotal shield absent. Setae *d1*, *d2*, and *e2* subequal in length. Pygidial shield present, well sclerotized and punctate. Setae *f2* 3.5–5 times longer than *f1*. Genital plate present, punctate. Pseudanal setae as microsetae. Length ratio of setae *ag1*:*ag2*:*ag3* 3.4:1:4. Coxal fields I–IV apunctate. Setae *3c* 1.3 times longer than *3b*. *Legs*. Setae *dFI*, *dGI*, *dTI*, *l'GI–IV*, *l'TI–IV*, and *l'RIII–IV* strongly knobbed, other leg setae slightly ornamented or smooth. *Lengths of setae*: *vi* 40 (40), *ve* 80 (70), *si* 95, *se* 110 (100), *c1* 140 (145), *c2* 115 (115), *d1* 110 (120), *d2* 115, *e2* 115 (120), *f1* 7 (7), *f2* 35 (25), *h1* 10, *h2* 260, *ag1* 120 (125), *ag2* 35, *ag3* 140 (130), *ps1* 5, *tc'III–IV* 30, *tc"III–IV* 70, *l'RIII* 35, *3b* 40, *3c* 50 (55), *4b* 40, *4c* 50 (55).

MALE, 2 paratypes. Characters as in female, except: total body length 350; stylophore 90 long; length ratios of setae *vi*:*ve*:*si* 1:1.5–1.6:2–2.2, *d2*:*d1*:*e2* 2.5:2:1; hysteronotal shield fused to pygidial shield, bearing bases of setae *d1*, *e2*, *f2* and *h2*; agenital plate well developed, bearing bases of setae *ag1*; setae *ag1* distinctly longer (6–9 times) than minute setae *ag2*; setae *3c* subequal to *3b* or setae *3c* 1.3 times longer than *3b*; lengths of setae: *vi* 20–25, *ve* 30–40, *si* 40–55, *se* 55, *c1* 55–60, *c2* 55, *d1* 20, *d2* 25, *e2* 10, *f2* 5, *ag1* 30–45, *ag2* 5, *l'RIII* 20–30, *l'RIV* 25, *3b* 20, *3c* 20–25, *4b* 20, *4c* 20–25.

**Type material.** Female holotype, 2 female and 2 male paratypes from the contour feather quill of Red and yellow Barbet *Trachyphonus erythrocephalus* Cabanis, 1878 (Piciformes: Lybiidae), **TANZANIA:** Arusha; 25 March 1960, coll. V. Nagy.

**Type material deposition.** All type material is deposited in the AMU (Reg. No. AMU-SYR.396).

### Key to genera of the subfamily Picobiinae

(Females)

1. Medial branch of peritremes with weakly marked borders between chambers. Movable cheliceral digit with 3 large teeth. Bases of setae *f2* situated far from *f1*. Opisthosomal lobes present. Antiaxial and paraxial claws of legs I and II dissimilar in shape and size … ***Calamincola* Casto, 1978**

–Medial branch of peritremes with well-marked borders between chambers. Movable cheliceral digit with 1–2 minute teeth. Bases of setae *f1* and *f2* situated in close proximity. Opisthosomal lobes absent. Antiaxial and paraxial claws of legs I and II similar in shape and size … **2**

2. Propodonotal shield entire or semi-divided with large medial shield. Physogastry weakly marked … ***Neopicobia*-generic-group** … **3**

–Propodonotal shield absent or distinctly divided into 2 narrow sclerites, medial shield present or absent. Physogastry well marked … ***Picobia*-generic-group** … **8**

3 Solenidion phi (*φ*) on tibia I absent … **4**

–Solenidion phi (*φ*) on tibia I present … ***Lawrencipicobia* Skoracki and Hromada, 2013**

4. Two pairs of pseudanal setae (*ps1*, *ps2*) present … **5**

–One pair of pseudanal setae (*ps1*) present … **6**

5. Genital setae absent … ***Neopicobia* Skoracki, 2011**

–One pair of genital setae (*g1*) present … ***Rafapicobia* Skoracki, 2011**

6. Lateral branch of peritremes with well-marked borders between chambers. Genital plate present … **7**

–Lateral branch of peritremes with weakly marked borders between chambers. Genital plate absent … ***Charadriineopicobia* Skoracki, Spicer and OConnor, 2014**

7. Propodonotal shield semi-divided with large medial shield bearing bases of setae *c1*. Setae *ve* situated in close proximity and postero- or posterolateral to *vi*. Setae *3a* smooth and thin. One pair of genital setae present (*g1*) … ***Pipicobia* Glowska and Schmidt, 2014**

–Propodonotal shield entire. Setae *ve* situated far and posteromedial to *vi*. Setae *ve* situated postero- or posterolateral to *vi*. Setae *3a* ornamented and thick. Genital setae absent … ***Tanopicobia* gen. nov.**

8. Pygidial shield and genital plate absent … **9**

–Pygidial shield and genital plate present … **11**

9. Medial shield of propodonotum bearing bases of setae *c1* … **10**

–Medial shield if present reduced to small sclerite not bearing bases of setae *c1* … ***Columbiphilus* Kivganov and Sharafat, 1995**

10. Peritremes V-shaped. Borders between chambers in lateral branch of peritremes well-marked. Bases of setae *vi* situated in close proximity to *ve*. Bases of setae *1a* coalesced. Two pairs of pseudanal setae present. Genital setae absent … ***Gunabopicobia* Skoracki and Hromada, 2013**

–Peritremes mouth-shaped. Borders between chambers in lateral branch of peritremes weakly marked. Bases of setae *vi* situated far from *ve*. Bases of setae *1a* not coalesced. Pseudanal setae absent. Two pairs of genital setae present … ***Phipicobia* Glowska and Schmidt, 2014**

11. Genital setae absent. Genital lobes absent … ***Pseudopicobia* Skoracki, Scibek and Sikora, 2012**

–One pair of genital setae present (*g1*). Genital lobes present … ***Picobia* Haller, 1878**

### Phylogenetic results

**Fig 3:**
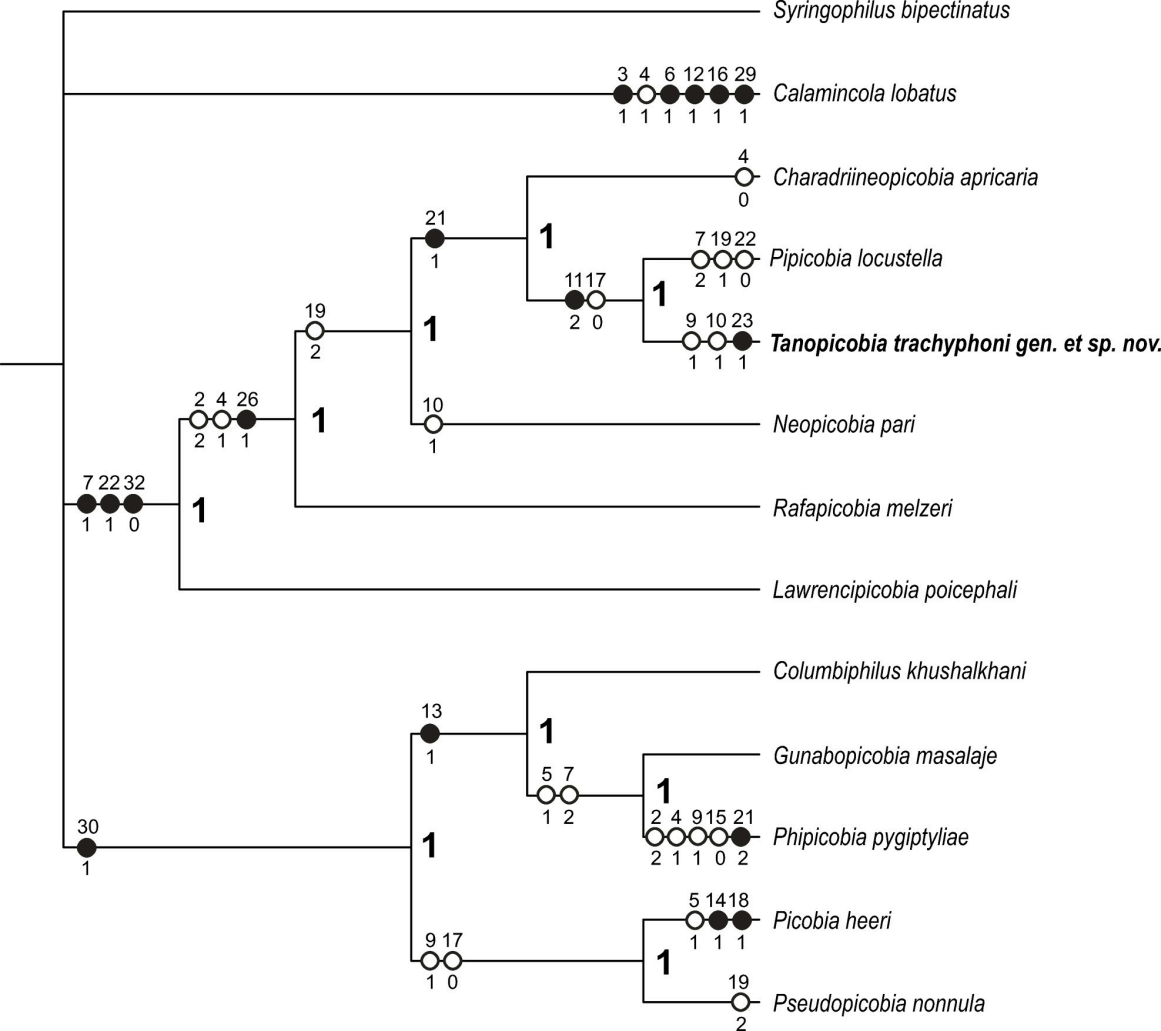
Strict consensus of two most parsimonious trees found for unordered and unweighted data set. Tree length 61, CI = 0.689, RI = 0.661, RC = 0.455, HI = 0.311 (uninformative characters included). Numbers above circles (black–unique apomorphy, white–homoplasy) refer to characters; numbers under circles refer to a character state achieved in the respective node. Numbers in ***Italic-bold*** near nodes are values of Bremer index.

The heuristic search produced two shortest trees having length 61 steps and standard indices as follows: CI = 0.689, RI = 0.661, RC = 0.455, HI = 0.311 (uninformative characters included). Strict consensus of these trees is shown in Fig 3.

**Fig 4:**
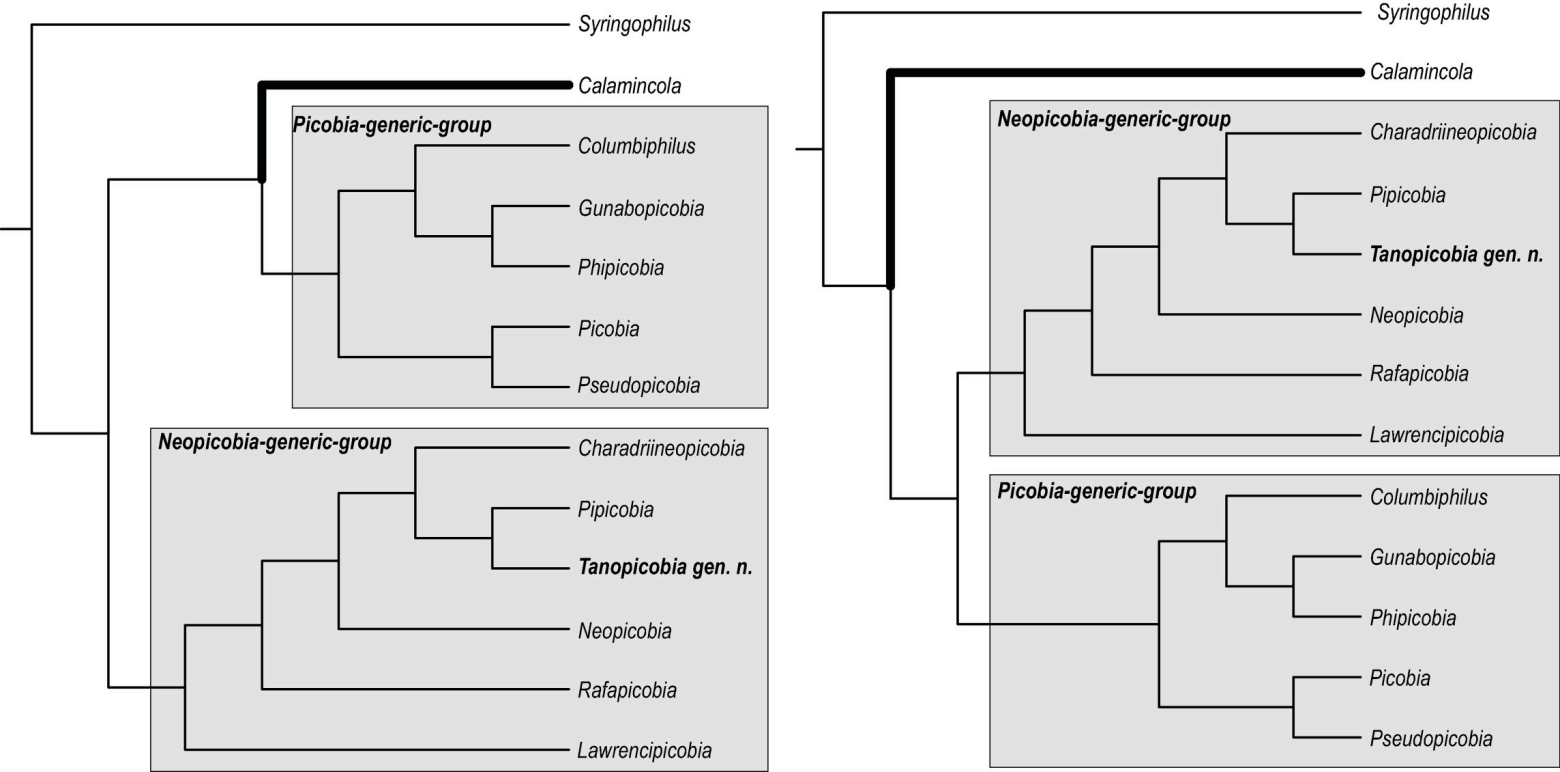
Position of the genus *Calamincola* in two most parsimonious trees.

The monophyly of subfamily Picobiinae seems to be obvious (see also [29]), but the position of the enigmatic genus *Calamincola* in relation to other genera is still questionable (Fig 4A and 4B). The analysis shows that all picobiine genera (excl. *Calamincola*) form two distinct clades: *Picobia*-generic-group including genera *Picobia*, *Pseudopicobia*, *Columbiphilus*, *Gunabopicobia*, *Phipicobia*, and supported by one synapomorphy (character 30) and clade *Neopicobia*-generic-group including the genera *Lawrencipicobia*, *Rafapicobia*, *Neopicobia*, *Charadriineopicobia*, *Pipicobia*, *Tanopicobia* gen. nov., and supported by three unique synapomorphies (characters 7, 22, 32).

The clade *Picobia*-generic-group is monophyletic and split into two clusters: cluster I–(*Gunabopicobia*+*Phipicobia*)+*Columbiphilus*, and cluster II–*Picobia+Pseudopicobia*. The *Neopicobia*-generic-group is monophyletic where the genus *Tanopicobia* is closely related to *Pipicobia*, with *Charadriineopicobia* as a sister group.

## Discussion

Based on phylogenetic analysis of the family Syringophilidae, the picobiines are placed in the core of the syringophilid tree, and the subfamily Syringophilinae is paraphyletic with respect to this group [29]. They are considerably more morphologically specialized than syringophilines and possess some advanced features like physogastry, which together with K reproductive strategy (few, but large eggs laid by a female [5]) probably allows them to occupy successfully small, but very numerous and always accessible quills of the contour (body) feathers [29]. Thus, picobiines are probably able to effectively avoid competition with other syringophilids and form an evolutionary line parallel to the syringophilines. Although it is possible to find 2–4 species of quill mites on one host, living in different types of feathers [5] and representing a different niches, there are no records of several picobiine species sharing one host; i.e., body feathers are always infested by only one picobiine species.

### Questionable placement of the genus *Calamincola*

The position of the enigmatic genus *Calamincola* remains unresolved.

In our study, the *Calamincola* represents either a sister group to all other picobiine genera (Fig 4A) or is a sister group to the genera forming *Picobia*-generic group (Fig 4B). It is worth to note that monoxenous *C*. *lobatus* represents the only species of the picobiine mite able to infest the quills of wing feathers, whereas all other genera occur entirely in contour feathers [1,3,4,24]. It is possible that the wing feathers, i.e., primaries and secondaries, are the ancestral type of syringophilid habitat, as the majority of the family Syringophilidae representatives are associated with the quills of these feathers [29–32]. Despite mites of the subfamily Picobiinae mostly dwell in the contour feathers, they possibly originally lived in wing quills [29]; thus, the archaic genus *Calamincola* could represent the "living fossil" among the syringophilid mites, however, this questions needs more in-depth investigation. It is obvious that the future research including molecular data is needed to resolve the position of this genus in the phylogenetic tree of syringophilid mites.

### Phylogeny and host-parasite relationships of the *Neopicobia*-generic group

**Fig 5:**
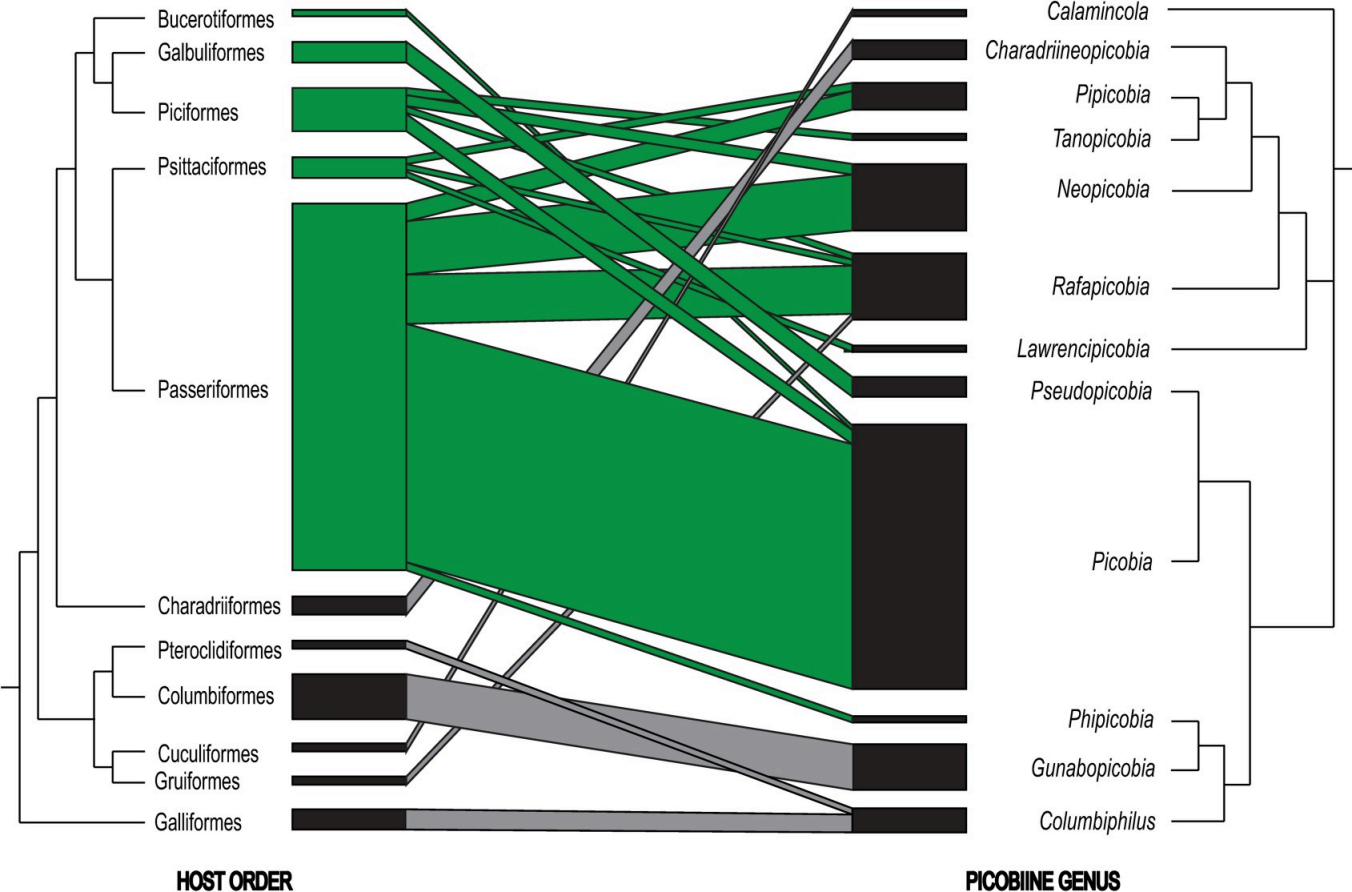
Host-parasite relationships between bird hosts (orders) and their picobiine parasites (genera). Host phylogeny based on Jetz et al. [21]. Interaction weight depicts a number of picobiine mite species parasitizing particular host order (Table 1). Bird orders included in Telluraves and their relationships with their ectoparasites in green.

This group of picobiine mites includes six genera: *Lawrencipicobia* + *Rafapicobia* + *Neopicobia* + *Charadriineopicobia* + (*Pipicobia* + *Tanopicobia*). Among them, three genera *Charadriineopicobia* (3 species), *Lawrencipicobia* (1), and *Tanopicobia* (1) parasitize birds of one order, Charadriiformes, Psittaciformes and Piciformes, respectively (Table 1 and Fig 5). The genus *Pipicobia* (4 species) is associated with birds of orders Passeriformes and Psittaciformes. Nowadays, there seems to be a general agreement that these two terminal clades are close, sister to each other [33–40]. On the other hand, *Neopicobia* (12 species) is associated with birds of the orders Passeriformes and Piciformes. Despite Piciformes were in pre-cladistic taxonomy considered to be a near-passerine taxon [41,42] and some early cladistic analyses also put them close to each other [43], today it is believed that they represent a different, rather distant lineage of Telluraves, core land birds [44]. This taxon comprises of two main clades: Afroaves, including the Piciformes, and Australaves, including Passeriformes and Psittaciformes [38–40,45,46].

Genus *Rafapicobia* (11 species) is associated with all three mentioned orders: Passeriformes, Psittaciformes, and Piciformes (Table 1 and Fig 5), thus, with both main clades of Telluraves. Moreover, one species of the genus, *R*. *melzeri* Skoracki et al., 2014, was also recorded on five host species of the rather distant family of rails (Gruiformes: Rallidae), which is outside Telluraves. Most probably, it is an example of a horizontal switch of picobiine parasites to phylogenetically distant hosts. To summarize, the host spectrum for *Neopicobia-*generic group cover advanced bird clades of telluriform birds plus neoavian Charadriiformes and Gruiformes (Table 1 and Fig 5). Despite these two bird taxa comprise well supported subclades in most recent phylogenomic studies [40,47,48], Reddy et al. [39] dubbed them, together with hoatzin (Opistomorphes), „orphaned”orders due to their uncler position within Neoaves. However, both are definitely rather distant from telluravian lineage. For a resolution to which extent host-parasite relationships in *Neopicobia-generic group* are a result of co-speciation with their host and/or which are host-switches, a more detailed investigation is needed.

### Phylogeny and host-parasite relationships of the *Picobia*-generic group

This group of picobiine mites includes five genera: (*Picobia* + *Pseudopicobia*) + (*Columbiphilus* + (*Gunabopicobia* + *Phipicobia*). Among them, three genera, *Gunabopicobia* (2 species), *Phipicobia* (1), and *Pseudopicobia* (3) are restricted to hosts of the one order, Columbiformes (Columbimorphae), Passeriformes, and Galbuliformes (both Telluraves), respectively (Table 1 and Fig 5). Genus *Columbiphilus* (4 species) is widely distributed on galliform birds (Galliformes, clade Galloanseres [37,38,46–50]), but one species, *C*. *pteroclesi* (Skoracki and OConnor, 2010) has been recorded on two host species of sandgrouse (Pterocliformes: Pteroclidae) [51]. Despite sandgrouses were formerly included in Galliformes due to their striking morphological and ecological similarities, later it was suggested that this is a result of convergent evolution [52]; recent phylogenies generally agree that they are not closely related to each other [37–40,47–50]. Thus, if current avian phylogenomic trees are correct, sharing the quill mite of genus *Columbiphilus* in these avian orders is interesting and probably an example of the horizontal transfer (Table 1 and Fig 5). Finally, the genus *Picobia* represents the most species-rich genus in the family Picobiinae (40 species). Representatives of this genus are associated with bird orders Passeriformes (clade Australaves) and Piciformes (Afroaves); moreover, one species, *P*. *phoeniculi* Fain et al. 2000, has been recorded on two avian species of the order Bucerotiformes (see Table 1 and Fig 5) (also Afroaves); thus, *Picobia* is well established on both main clades of Telluraves [37,45].

To conclude, the most ancestral avian lineage hosting Picobiinae are Galliformes on Galloanseres, a basal Neognathae clade, which split from the rest of neoavian birds around 85 Mya, before Cretaceous-Paleogene (C-Pg) transition [40,48,50]. The root of Neoaves, which radiated very quickly in the period close to C-Pg, stays mostly unresolved despite several recent advanced whole-genome reconstructions [37–40,47,49,50,53]. Picobiine mites are present in Columbimorphae clade, containing Columbiformes and Pterocliformes; also their quill mites seem to be closely related (Fig 5). Another basal neoavian lineage parasitized with picobiine mites is Cuculiformes (clade Otidimorphae [38,40,44,46–49]), infested with *Calamincola* with unclear status. The last lineages hosting Picobiinae mites outside Telluraves are Gruiformes and Charadriiformes, which are probably close, sister to each other [37,50] (but see [39]); our mite phylogeny (Fig 5) is also not in conflict with this topology.

However, the most extensive radiation of Picobiinae mites took place in megadiverse terminal clade Telluraves (Fig 5), well supported by all recent bird phylogenies [37–40,44–50]. In general, syringophilid mites are rather highly host specific [1,7]; thus, the extent to which observed relationships are the result of co-speciation and where host switches occurred needs another, more detailed analyses, as well as more robust trees of both quill mites and their avian hosts.

For the future, the phylogenies based both on mite morphological and molecular data are needed. Such analyses can be used to group the numerous picobiine (and syringophilid in general) genera described to date and provide a solid basis for the analysis of the co-evolutionary relationships between these parasitic mites and their bird hosts. There are, however, two main problems, which complicate co-evolutionary reconstructions of the picobiine relationship with their hosts. Firstly, subfamily is rather uniform morphologically, as it possesses only a limited set of the external morphological structures [1]. They are represented mostly by setae, where the combination of traits such as the presence/absence of particular setae are the main generic characteristics. Such features have a high probability of being of homoplastic origin and are, therefore, less reliable in phylogenetic analyses (see also [28]). Secondly, material suitable for molecular analyses is absent for most picobiine taxa (which is true also for syringophilids as a whole) as the main body of quill mite species descriptions come from old bird skins in museum collections [54]; therefore, there are certain problems with the ancient DNA isolation from the material [55,56,57]. We are aware of the flaws of our morphological approach, but we believe that our data will be helpful as a background for future molecular-based studies as they can provide useful diagnostic synapomorphies [58].

## Supporting information

S1 Table Data matrix of character states for Picobiinae and outgroup taxa. Character states are scored as 0 to 3, inapplicable states as "-", missing states as "?".
(DOCX)

S2 Table Characters used in the phylogenetic analysis. (DOCX)
