# Supplementary material for: Tanopicobia gen. nov., a new genus of quill mites, its phylogenetic placement in the subfamily Picobiinae (Acariformes: Syringophilidae) and picobiine relationships with avian hosts
Source: PLoS One. 2020 Jan 15;15(1):e0225982. doi: 10.1371/journal.pone.0225982 (PMC6961858; doi:10.1371/journal.pone.0225982)
Supplement: S1 Table — Character states are scored as 0 to 3, inapplicable states as "-", missing states as "?". (DOCX) [file pone.0225982.s001.docx]

**S1 Table. Data matrix of character states for Picobiinae and outgroup taxa.** Character states are scored as 0 to 3, inapplicable states as "-", missing states as "?".

| **Taxa** | **Character states** |
| --- | --- |
|  | 11111111112222222222333 |
|  | 12345678901234567890123456789012 |
| *Syringophilus bipectinatus* Heller, 1880 | 0000000000000000100000000000000- |
| *Calamincola lobatus* Casto, 1977 | 101101300011?011?010000110111011 |
| *Charadriineopicobia apricaria* Skoracki et al. 2016 | 1200201000100000102-110111110010 |
| *Columbiphilus khushalkhani* Kivganov and Sharafat, 1995 | 11000030001010101010000110110111 |
| *Gunabopicobia masalaje* Kaszewska et al., 2014 | 1100102000101010102-000110110111 |
| *Lawrencipicobia poicephali* (Skoracki and Dabert, 2002) | 11003010001000001012010110110?10 |
| *Neopicobia pari* Skoracki et al. 2016 | 1201201001100000102-010111110010 |
| *Phipicobia pygiptilae* Glowska and Schmidt, 2014 | 120110201010100010002-0110110?11 |
| *Picobia heeri* Haller, 1878 | 110010311-1001100111000110110111 |
| *Pipicobia locustella* (Skoracki et al., 2004) | 12012020002000000010100111110010 |
| *Pseudopicobia nonnula* Skoracki et al, 2012 | 110000321-100010002-000110110?11 |
| *Rafapicobia melzeri* Skoracki et al., 2014 | 12012010001000001012010111110010 |
| *Tanopicobia* *trachyphoni* gen. n. et sp. n. | 1201201011200000002-111111110?10 |
