# Supplementary material for: Tanopicobia gen. nov., a new genus of quill mites, its phylogenetic placement in the subfamily Picobiinae (Acariformes: Syringophilidae) and picobiine relationships with avian hosts
Source: PLoS One. 2020 Jan 15;15(1):e0225982. doi: 10.1371/journal.pone.0225982 (PMC6961858; doi:10.1371/journal.pone.0225982)
Supplement: S2 Table — (DOCX) [file pone.0225982.s002.docx]

**S2 Table.** **Characters used in the phylogenetic analysis.**

| **No** | **Characters and coding** |
| --- | --- |
|  | Gnathosoma |
| 1 | Tibiotarsus of palp rounded (**0**), tapering (**1**) |
| 2 | Peritremes U-shaped (**0**), V- or M-shaped (**1**), mouth-shaped (**2**) |
| 3 | Chambers in medial branch of peritremes well developed (**0**), poorly developed (**1**) |
| 4 | Chambers in lateral branch of peritremes well developed (**0**), poorly developed (**1**) |
| 5 | Hypostomal apex rounded (**0**), hilly (**1**), tapering (**2**), flat (**3**) |
| 6 | Movable cheliceral digit with 1–2 minute teeth (**0**), with 3 large teeth (**1**) |
|  | Idiosoma |
| 7 | Propodonotal shield entire rectangular in shape (**0**), entire M-shaped (**1**), semi-divided with large medial plate with bases of setae *c1* (**2**), distinctly divided without medial shield or with small medial shield not bearing setal bases *c1* (**3**) |
| 8 | Bases of setae *vi* situated anterior to *ve* (**0**), *vi* and *ve* at same level (**1**), *vi* posterior to *ve* (**2**) |
| 9 | Bases of setae *vi* and *ve* situated in close proximity (**0**), *ve* situated far from *vi* (**1**) |
| 10 | Bases of setae *ve* situated postero- or posterolateral to *vi* (0), *ve* posteromedial to *vi* (1) |
| 11 | Bases of setae *d1* situated closer to *d2* than to *e2* (**0**), *d1* in equidistant to *d2* and *e2* (**1**), *d1* closer to *e2* than to *d2* (**2**) |
| 12 | Bases of setae *f1* and *f2* situated in close proximity (**0**), setae *f2* situated far from *f1* (**1**) |
| 13 | Pygidial shield present (**0**), absent (**1**) |
| 14 | Pygidial lobes absent (**0**), present (**1**) |
| 15 | Bases of setae *1a* not coalesced (**0**), coalesced (**1**) |
| 16 | Opisthonotal lobes absent (**0**), present (**1**) |
| 17 | Genital plate present (**0**), absent (**1**) |
| 18 | Genital lobes absent (**0**), present (**1**) |
| 19 | Genital setae: 2 pairs (**0**), 1 pair (**1**), absent (**2**) |
| 20 | Genital setae setiform (**0**), rod-like (**1**), as microsetae (**2**) |
| 21 | Pseudanal setae: 2 pairs (**0**), 1 pair (**1**), absent (**2**) |
| 22 | Pseudanal setae setiform (**0**), as microsetae (**1**) |
| 23 | Setae *3a* thin and smooth (**0**), thick and ornamented (**1**) |
|  | Legs |
| 24 | Leg setae *p'* and *p"* fan-like (**0**), rod-like (**1**) |
| 25 | Leg setae *dFIII-IV* present (**0**), absent (**1**) |
| 26 | Solenidion *phi* present (**0**), absent (**1**) |
| 27 | Apodemes I and II fused to each other (**0**), not fused (**1**) |
| 28 | Setae *l'RI-II* present (**0**), absent (**1**) |
| 29 | Antiaxial and paraxial claws of legs I and II similar in shape (**0**), dissimilar in shape (**1**) |
| 30 | Claws I and II without claw-wings (**0**), with claw-wings (**1**) |
|  | Physogastry |
| 31 | Physogastry absent (**0**), present (**0**) |
| 32 | Physogastry weakly-marked (**0**), well-marked (**1**) |
